# Do Exercise‐Induced Extracellular Vesicles Carry Cognitive Benefits to the Brain?

**DOI:** 10.1111/tra.70046

**Published:** 2026-07-24

**Authors:** Oliver K. Fuller, Thierry Galli

**Affiliations:** ^1^ School of Life and Environmental Sciences, Faculty of Science The University of Sydney Sydney New South Wales Australia; ^2^ Charles Perkins Centre The University of Sydney Sydney New South Wales Australia; ^3^ Université Paris Cité, Institute of Psychiatry and Neuroscience of Paris, INSERM U1266, Membrane Traffic in Healthy & Diseased Brain Paris France; ^4^ GHU Paris Psychiatrie & Neurosciences Paris France

## Abstract

Exercise‐induced muscle‐brain communication mediated by extracellular vesicles. Physical exercise stimulates skeletal muscle to release extracellular vesicles (EVs) into the circulation. These vesicles may reach the brain and transfer bioactive cargo such as miRNA, thereby supporting neuronal function and brain homeostasis and potentially protecting against the onset or progression of neurodegenerative diseases.

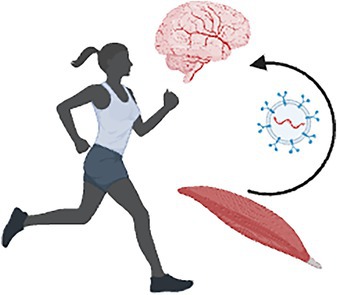

Physical exercise is among the most robust non‐pharmacological interventions known to delay cognitive decline and reduce the risk of Alzheimer's disease. Yet the mechanistic basis of this protection remains incompletely understood. In a recent study in *Nature Aging*, Lin and colleagues propose an intriguing solution to this long‐standing question: extracellular vesicles (EVs) released by skeletal muscle during exercise may function as systemic signaling organelles capable of crossing the blood–brain barrier and reprogramming microglial function in the diseased brain [1].

The study places membrane trafficking and inter‐organ communication at the center stage. Using exercise paradigms in APP/PS1 mice, the authors show that intense swimming enhances the secretion of skeletal muscle‐derived EVs (SKM‐EVs), which accumulate in brain microglia and promote disease‐associated microglial activation and amyloid plaque clearance. Particularly compelling is the use of muscle‐specific CD63‐GFP reporter mice, which provides evidence that vesicular material originating from skeletal muscle can ultimately be detected in the cortex and hippocampus. This peripheral‐to‐brain trafficking axis represents a major conceptual advance for the EV field and reinforces the emerging idea that systemic physiological states can be encoded through vesicular communication [2, 3].

The work is also notable for its breadth. The authors combine exercise physiology, behavioral analyses, EV transfer experiments, microglial depletion, muscle‐specific Dicer deletion, miRNA profiling, and engineered vesicle delivery approaches into a remarkably coherent experimental framework. The convergence of gain‐ and loss‐of‐function strategies strongly supports a causal contribution of exercise‐induced vesicular signaling to the observed cognitive benefits.

At the same time, the study illustrates some of the persistent conceptual and technical challenges that continue to accompany EV biology.

A first set of considerations concerns the quantitative scope of the proposed mechanism. Recent work using dual‐fluorescent reporter mice has shown that skeletal muscle myofiber‐derived EVs, despite being secreted in abundance ex vivo, account for only approximately 4%–5% of circulating tetraspanin‐positive EVs under free‐living conditions, with very limited detection in plasma by spectral flow cytometry [4]. Reconciling this relatively modest contribution to the circulating EV pool with the robust cognitive effects reported by Lin and colleagues will be an important next step. Related to this, the demonstration of GFP‐positive signals in the CNS after muscle‐specific CD63 labeling is elegant and provocative, but fluorescence transfer does not necessarily establish that intact vesicles themselves cross the blood–brain barrier. Alternative possibilities remain plausible, including endothelial uptake and relay mechanisms, peripheral immune intermediates, or partial cargo transfer rather than translocation of complete vesicles. Similarly, the uptake mechanism by microglia has been only superficially explored and is largely attributed to pinocytosis. Defining the cellular itinerary of SKM‐EVs—from muscle secretion to brain entry and microglial targeting—will be essential if these pathways are to become therapeutically exploitable.

Methodological choices around EV preparation also deserve attention. The authors rely on sequential differential ultracentrifugation as their sole isolation strategy. While this is a widely used approach, it is known to co‐isolate protein aggregates, lipoproteins, and other non‐vesicular nanoparticles, and the field increasingly recommends orthogonal validation through size‐exclusion chromatography, density‐gradient separation, or immunoaffinity capture. Incorporating such complementary methods would strengthen confidence that the observed biological effects are attributable to bona fide vesicular signaling rather than to co‐purifying material. In a similar vein, the pharmacological blockade of EV secretion with GW4869, even when elegantly targeted to muscle via the RGDLTTP‐modified liposome, primarily inhibits ceramide‐dependent exosome biogenesis and does not efficiently block microvesicle release from the plasma membrane [5], a pathway known to be activated during exercise [6]. The near‐complete abrogation of exercise benefits under this single‐pathway inhibition is therefore intriguing, though further dissection will be needed to determine which vesicle subtypes are functionally responsible.

A related issue concerns the choice of swimming as the exercise paradigm. Forced swimming, while well established as an aerobic stimulus, is also a potent psychological stressor in rodents and is known to engage HPA‐axis and inflammatory responses that may themselves modulate microglial state independently of muscle‐derived signaling. Disentangling exercise‐specific from stress‐related contributions remains a recurring challenge in this literature, and complementary paradigms such as voluntary wheel running could help anchor the conclusions.

Perhaps the most debatable aspect of the study is the progressive reduction of the exercise phenotype to a single miRNA cargo, miR‐378a‐3p. Through Dicer knockout models, miRNA sequencing, agomirs, AAV‐mediated manipulations, and engineered EVs, the authors build an impressive argument implicating this miRNA in the beneficial effects of exercise. Yet the biological interpretation may risk becoming overly reductionist. Exercise induces profound systemic metabolic, endocrine, vascular, inflammatory, and neuronal changes, including increased cerebral blood flow, hippocampal neurogenesis, BDNF and irisin signaling, and broad anti‐inflammatory effects [7], and EVs themselves carry highly complex molecular repertoires including proteins, lipids, metabolites, and multiple RNA species. It therefore seems unlikely that the cognitive and immunological consequences of exercise can be fully captured by a single vesicular miRNA. The authors themselves note that they cannot exclude contributions from other EV cargo, and the apparent completeness of these phenotypic effects may reflect the limited sensitivity of the APP/PS1 model to other parallel pathways, or a convergence of multiple exercise mechanisms onto shared microglial endpoints. Either possibility raises a broader conceptual challenge for the field: should EVs be viewed as discrete carriers of individual instructive molecules, or rather as integrated multimolecular signaling platforms whose function emerges from combinatorial cargo composition? The answer will likely determine how future therapeutic EV engineering strategies are designed.

Despite these caveats, the study by Lin et al. substantially advances the growing view that vesicular trafficking participates in organism‐wide physiological coordination. The notion that skeletal muscle can remotely influence brain immunity through EV‐mediated communication is both exciting and provocative. More broadly, the work highlights how peripheral tissues may dynamically shape CNS homeostasis through trafficking pathways that remain only partially understood.

For the trafficking community, this study provides an important reminder: vesicles are not merely cellular waste containers or biomarkers, but potentially systemic vectors of physiological state. Understanding precisely what these vesicles are, how they travel, and what information they truly carry will now become the next critical challenge.

## Funding

This work was supported by Agence Nationale de la Recherche, ANR‐18‐IDEX‐0001.

## Conflicts of Interest

The authors declare no conflicts of interest.

## Data Availability

The data that support the findings of this study are available on request from the corresponding author. The data are not publicly available due to privacy or ethical restrictions.
